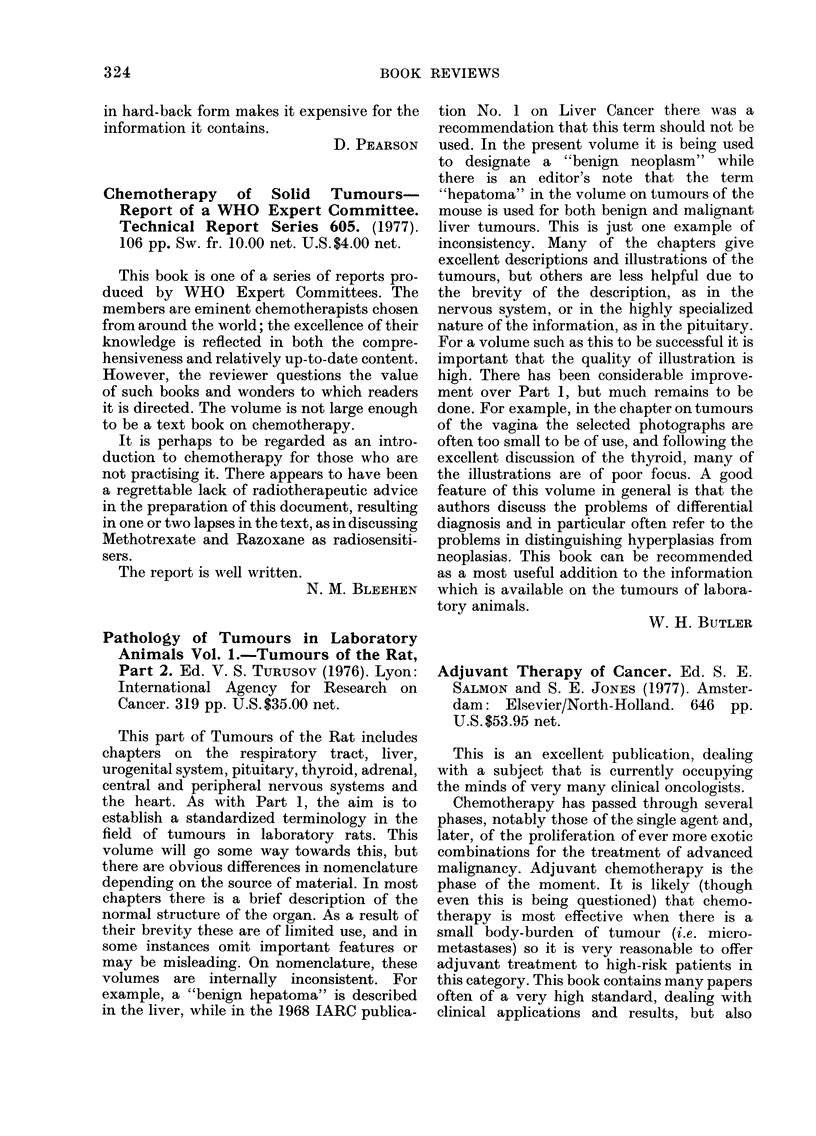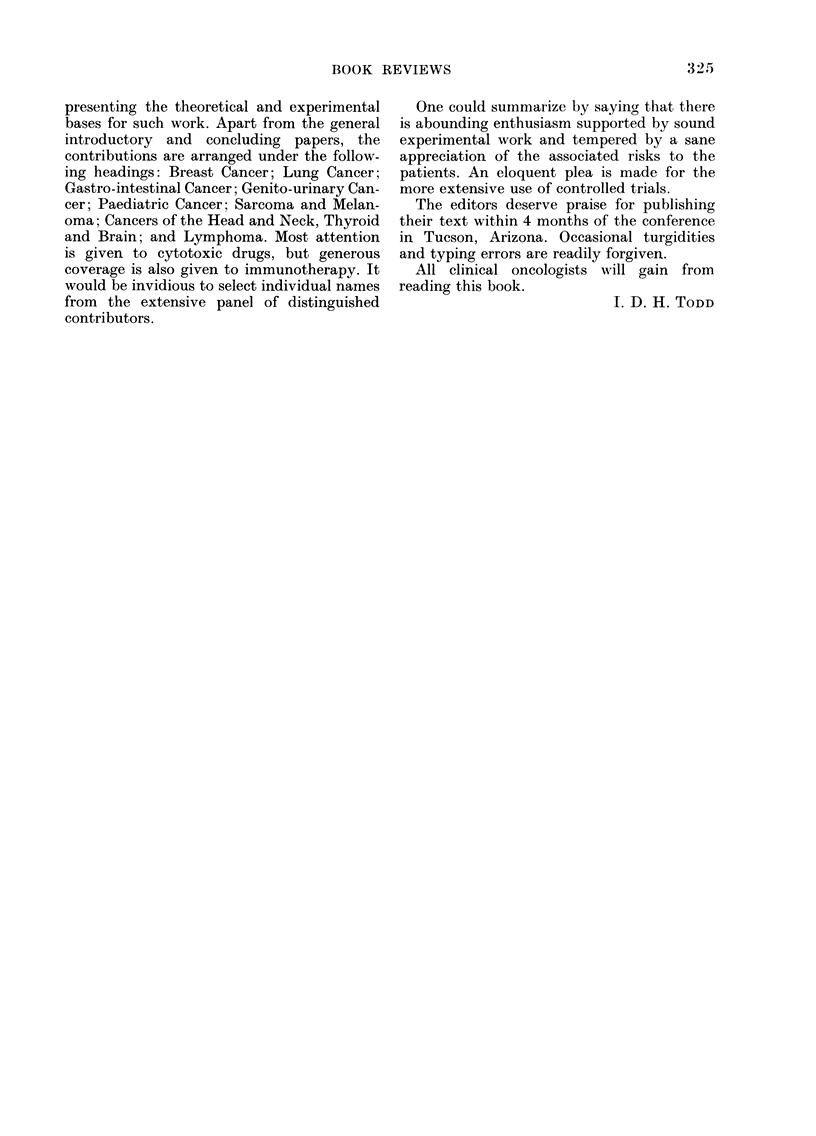# Adjuvant Therapy of Cancer

**Published:** 1978-02

**Authors:** I. D. H. Todd


**Adjuvant Therapy of Cancer.** Ed. S. E. SALMON and S. E. JONES (1977). Amsterdam: Elsevier/North-Holland. 646 pp. U.S.$53.95 net.

This is an excellent publication, dealing with a subject that is currently occupying the minds of very many clinical oncologists.

Chemotherapy has passed through several phases, notably those of the single agent and, later, of the proliferation of ever more exotic combinations for the treatment of advanced malignancy. Adjuvant chemotherapy is the phase of the moment. It is likely (though even this is being questioned) that chemotherapy is most effective when there is a small body-burden of tumour (*i.e.* micrometastases) so it is very reasonable to offer adjuvant treatment to high-risk patients in this category. This book contains many papers often of a very high standard, dealing with clinical applications and results, but also presenting the theoretical and experimental bases for such work. Apart from the general introductory and concluding papers, the contributions are arranged under the following headings: Breast Cancer; Lung Cancer; Gastro-intestinal Cancer; Genito-urinary Cancer; Paediatric Cancer; Sarcoma and Melanoma; Cancers of the Head and Neck, Thyroid and Brain; and Lymphoma. Most attention is given to cytotoxic drugs, but generous coverage is also given to immunotherapy. It would be invidious to select individual names from the extensive panel of distinguished contributors.

One could summarize by saying that there is abounding enthusiasm supported by sound experimental work and tempered by a sane appreciation of the associated risks to the patients. An eloquent plea is made for the more extensive use of controlled trials.

The editors deserve praise for publishing their text within 4 months of the conference in Tucson, Arizona. Occasional turgidities and typing errors are readily forgiven.

All clinical oncologists will gain from reading this book.

I. D. H. Todd